# Comparison of short-term outcomes between robotic and laparoscopic liver resection: a meta-analysis of propensity score-matched studies

**DOI:** 10.1097/JS9.0000000000000857

**Published:** 2023-11-03

**Authors:** Fengwei Gao, Xin Zhao, Qingyun Xie, Kangyi Jiang, Tianyang Mao, Manyu Yang, Hong Wu

**Affiliations:** a Liver Transplantation Center, State Key Laboratory of Biotherapy and Cancer Center, West China Hospital, Sichuan University and Collaborative Innovation Center of Biotherapy, Chengdu; b Department of Hepato-Pancreato-Biliary Surgery, The People’s Hospital of Leshan, Leshan; c North Sichuan Medical College, Nanchong, Sichuan, People’s Republic of China

**Keywords:** laparoscopy, liver resection, meta-analysis, propensity score matching, robot

## Abstract

**Objective:**

This meta-analysis aimed to compare short-term outcomes between robotic liver resection (RLR) and laparoscopic liver resection (LLR) using data collected from propensity score-matched studies.

**Methods:**

The PubMed, Cochrane Library, and Embase databases were searched to collect propensity score-matched studies comparing RLR and LLR. Relevant data were extracted and analyzed. Odds ratios (ORs) and standardized mean differences (SMDs) with 95% confidence intervals (CIs) were calculated using fixed-effect or random-effect models. Meta-regression analysis was performed for primary outcome measures. Subgroup analyses and sensitivity analyses were performed for outcomes exhibiting high heterogeneity. Quality of evidence was evaluated using the Grading of Recommendations, Assessment, Development and Evaluation framework.

**Results:**

Twenty-two propensity score-matched studies were included to comprise 5272 patients (RLR group, 2422 cases; LLR group, 2850 cases). Intraoperative blood loss (SMD=−0.31 ml, 95% CI −0.48 to −0.14; *P*=0.0005), open conversion (OR=0.46, 95% CI 0.37–0.58; *P* <0.0001), and severe complications (OR=0.76, 95% CI 0.61–0.95; *P*=0.02) were significantly lower in the RLR group. Operation time, odds of use, and duration of Pringle maneuver, length of hospital stay, and odds of intraoperative blood transfusion, overall complications, R0 resection, reoperation, 30-day readmission, 30-day mortality, and 90-day mortality did not significantly differ between the groups. Further subgroup and sensitivity analyses suggested that the results were stable. Meta-regression analysis did not suggest a correlation between primary outcomes and study characteristics. The quality of evidence for the primary outcomes was medium or low, while that for the secondary outcomes was medium, low, or very low.

**Conclusion:**

Although some short-term outcomes are similar between RLR and LLR, RLR is superior in terms of less blood loss and lower odds of open conversion and severe complications. In the future, RLR may become a safe and effective replacement for LLR.

## Introduction

Highlights

- The first meta-analysis of propensity score-matched studies to compare short-term outcomes between robotic liver resection (RLR) and laparoscopic liver resection (LLR).
- The largest number of included studies, the largest sample size comparison RLR and LLR meta-analysis.
- RLR is superior in terms of less blood loss and lower odds of open conversion and severe complications.

Reich *et al*.^1^ first reported laparoscopic hepatectomy in 1991. Laparoscopic hepatectomy is associated with less intraoperative bleeding, lower incidence of postoperative complications, shorter hospital stay, and faster recovery than open hepatectomy and is widely used around the world^2–4^. Laparoscopic hepatectomy has several limiting factors including limited angles of movement for instruments, two-dimensional visual field, poor stability, poor ergonomics, and dependence on assistants. Furthermore, liver surgery is quite complex and variable and requires intraoperative adaptability^5–7^. The emergence of robotic surgery has overcome these shortcomings to some extent, as it can provide increased instrument range of motion, three-dimensional visualization, and better stability and ergonomics^8,9^.

Several previous studies have confirmed the safety and feasibility of robotic hepatectomy as well as its advantages over open hepatectomy^10–12^. However, whether robotic hepatectomy is superior to laparoscopic hepatectomy is controversial. In a matched comparison of robotic liver resection (RLR) and laparoscopic liver resection (LLR) cases, Tsung *et al*.^13^ reported that both were similar in terms of safety and feasibility. Chong *et al*.^14^ compared robotic and laparoscopic right hepatectomy in a propensity score-matched analysis and found that the open conversion rate was lower and the hospital stay was shorter in the robotic cases. Two other studies have reported only that RLR was associated with less blood loss than LLR^15,16^. In contrast, a recent propensity score-matched analysis of patients with large hepatocellular carcinomas found no significant difference in perioperative results^17^. These results suggest that higher quality studies are needed to determine whether RLR or LLR is superior.

To the best of our knowledge, no randomized controlled trial (RCT) has directly compared RLR and LLR; however, meta-analyses have been conducted^18–23^. One reported that LLR was associated with less bleeding and shorter operation time than RLR, while two others found no such differences^18,20,22^. The meta-analyses reported to date have several problems: the numbers of studies and patients included were small and studies using propensity score matching were not included. Therefore, their results are not very reliable. More recently, studies have been comparing RLR and LLR using propensity score matching. We present a meta-analysis of these studies to compare short-term outcomes between RLR and LLR.

## Methods

### Search strategy

Based on systematic review and meta-analysis (PRISMA) and assessment of the methodological quality of systematic review (AMSTAR), it is carried out in the south^24,25^. The study was registered in PROSPERO. We searched the PubMed, Embase, and Cochrane Library databases to collect propensity score-matched studies that compared short-term outcomes between RLR and LLR published before 30 April 2023. Key search words included the following: robot, robotic, laparoscopy, laparoscopic, liver resection, hepatectomy, sectionectomy, propensity score-matched, and propensity score matching. Search strategies are provided in detail in Supplemental Tables S1–S3 (Supplemental Digital Content 1, http://links.lww.com/JS9/B268). References in studies identified were also searched to identify other potential studies that met the criteria.

### Inclusion and exclusion criteria

The Population, Intervention, Comparator, and Outcomes model was used to determine the inclusion criteria: population − all patients who underwent hepatectomy; intervention − RLR; comparator − LLR; outcomes − operation time, intraoperative blood loss volume, conversion to laparotomy, and length of hospital stay. In addition, we only included studies published in English and those with Newcastle–Ottawa scale (NOS) score >5. Abstracts, case reports, reviews, and studies that had a matched cohort but did not use the tendency score method to match were excluded.

When there was a queue of multiple score matching ratios, data were prioritized in the following order of RLR:LLR ratios (1:1>1:2>1:3). The most recent published data were used when there were multiple related studies by the same author. Larger sample sizes were used when there were studies by the same author at the same time.

### Data extraction

Three researchers extracted data according to the extraction form and reviewed it. Inconsistencies and disputes were resolved by discussion with a fourth researcher who made the decision. General data extracted included the following: article title, author, publication date and country, number of patients, male-to-female ratio, age, and other clinical data. Primary outcomes data included overall complications, severe complications (Clavien–Dindo grade II and higher), and R0 resection rate. Secondary outcomes included operation time, intraoperative blood loss volume, application and duration of Pringle maneuver, rate of conversion to laparotomy, intraoperative blood transfusion, length of hospital stay, reoperation rate, 30-day readmission rate, and 30-day and 90-day mortality rates.

### Quality assessment

Study quality was evaluated by two researchers using the NOS. Any differences in evaluation were evaluated by a third researcher and resolved through negotiation.

### Statistical analysis

Statistical analyses were performed using RevMan software version 5.4 (Cochrane, London, UK). Binary short-term outcomes were evaluated using odds ratios (ORs) with 95% confidence intervals (CIs); continuous outcomes were evaluated using standardized mean differences (SMDs) with 95% CIs. We used the method proposed by Hozo *et al*.^26^ to estimate only the mean and standard deviation of the median, extreme value, and quartile spacing. The *Q* test and heterogeneity statistic (*I*^2^) were used to evaluate study heterogeneity. When *I*^2^ was less than 50%, the fixed-effects model was used for analysis; when it was greater than or equal to 50%, the random-effects model was used^27^.

Meta-regression analysis was performed using Stata software version 14 (StataCorp, College Station, Texas, USA) to explore the relationship between primary outcomes and patient characteristics. The variables considered included publication year, percentage of men, average age, sample size, American Society of Anesthesiologists (ASA) physical status grade, and study NOS score. For results with high heterogeneity (*I*^2^ >50%), a leave-one-out meta-analysis of sensitivity score and subgroup analyses according to publication time, number of participating centers, and NOS score were performed using RevMan software version 5.4. Publication bias was evaluated using a funnel chart. All statistical tests were two-sided. *P* <0.05 was considered significant.

GRADE Pro software version 3.6 was used to evaluate the quality of evidence for outcomes. Quality was rated as high, medium, low, or very low based on research design, research quality, accuracy, consistency, directness, and risk of reporting bias.

## Results

### Study selection

**Figure 1:**
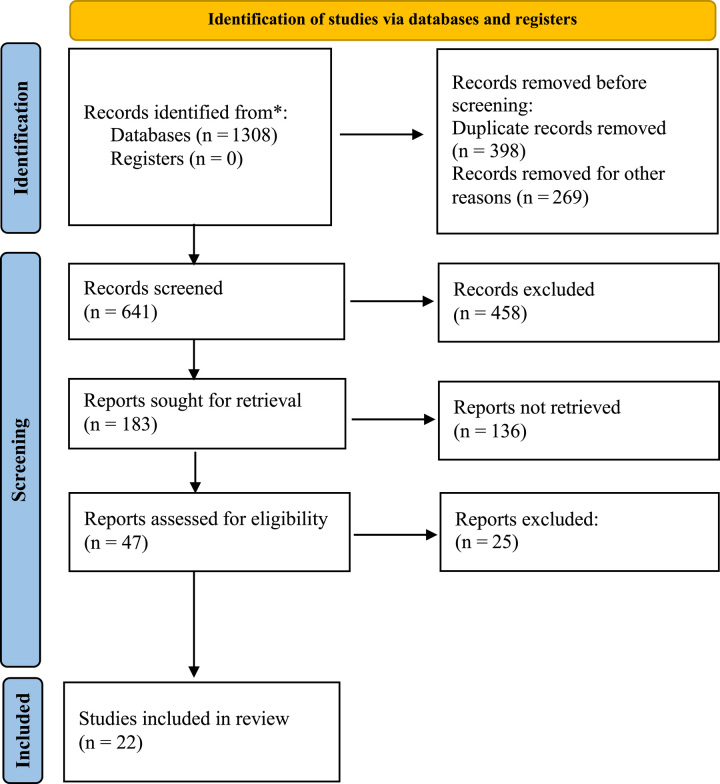
PRISMA diagram for the selection of the studies.

After identifying 641 potential articles, 22 propensity score-matched studies were included for analysis^14–17,28–45^. Among these, 12 were multicenter international studies^14–17,30,31,34,37,38,40,42,43^, 10 were from the International Robotic and Laparoscopic Liver Resection Study Group collaborators^14–17,34,37,38,40,42,43^, and one was a living donor hepatectomy study^36^. A total of 5272 patients (2422 who underwent RLR and 2850 who underwent LLR) were analyzed. The study flow chart is shown in Fig. 1. Details and NOSs of the included studies are shown in Table 1 and Supplemental Table S4 (Supplemental Digital Content 2, http://links.lww.com/JS9/B270).

**Table 1:** General characteristics of the included studies.

First author	Year	Country	Surgery	Cases (*n*)	Age (year)	Sex (male, *n*)	ASA score (I–II/III–IV, *n*)	BMI (kg/m^2^)	CRLM/HCC/other (*n*)	Median tumor size (cm)	Extent of resection
Montalti *et al*.^28^	2016	MC	RLR	36	62±13	21	23/13	N	21/3/12	4.44±3.06	S7, S8, S4a, S1
			LLR	72	56.8±15	39	52/20	N	44/6/22	4.95±3.5	
Salloum *et al*.^29^	2016	France	RLR	14	57±12	N	10/4	N	N	N	N
			LLR	14	57±15	N	10/4	N	N	N	
Lim *et al*.^30^	2019	MC	RLR	55	65±10	37	N	25±4	13/38/4	4.0±2.4	N
			LLR	55	66±10	41	N	27±6	11/36/8	4.0±2.4	
Beard *et al*.^31^	2020	MC	RLR	115	61±11	76	21/94	28±6	115/0/0	N	N
			LLR	115	61±12	75	16/99	29±6	115/0/0	N	
Chiow *et al*.^15^	2021	MC	RLR	88	60 (51–69)	59	52/36	N	21/52/15	3.5 (3–5)	Right posterior sectionectomy
			LLR	88	61 (54–69)	64	56/32	N	21/54/13	4 (3–5.2)	
Fagenson *et al*.^32^	2021	USA	RLR	240	60 (50–69)	142	N	27.9(24.3–32.7)	88/64/88	N	N
			LLR	240	63 (51–73)	140	N	27.6(24.0–32.0)	88/64/88	N	
Chong *et al*.^14^	2022	MC	RLR	220	61 (52–69)	139	133/87	N	57/106/57	5 (3–7)	Right and extended right hepatectomy
			LLR	220	61 (55–71)	144	128/92	N	59/104/57	5 (3–7.5)	
Cipriani *et al*.^33^	2022	Italy	RLR	288	N	168	164/124	N	77/115/96	N	N
			LLR	864	N	493	486/378	N	216/307/341	N	
D’Silva *et al*.^16^	2022	MC	RLR	104	62 (53–68)	70	64/40	N	30/54/20	2.5 (1.6–3.5)	Posterosuperior segments
			LLR	104	63 (50–70)	68	64/40	N	30/54/20	2.5 (1.8–3.5)	
Kadam *et al*.^34^	2022	MC	RLR	296	61 (52–67)	191	173/123	N	58/155/83	2.6 (2.0–4.0)	N
			LLR	296	61 (51–70)	196	173/123	N	58/155/83	2.7 (1.8–4.0)	
Kamel *et al*.^35^	2022	USA	RLR	182	N	N	N	N	N	N	N
			LLR	182	N	N	N	N	N	N	
Rho *et al*.^36^	2022	USA	RLR	19	29.3±10.5	13	N	22.4±2.1	N	N	Right hepatectomy
			LLR	19	30.3±11.1	11	N	21.9±2.1	N	N	
Sucandy *et al*.^37^	2022	MC	RLR	164	62±17.3	100	104/60	N	32/69/63	4.65±3.0	Left and extended left hepatectomy
			LLR	164	63±15	105	101/63	N	30/66/68	4.1±4.28	
Yang *et al*.^38^	2022	MC	RLR	40	62 (55–68)	32	29/11	N	7/25/8	3.8 (3.0–4.9)	Right anterior sectionectomy and central hepatectomy
			LLR	40	62 (54–72)	33	27/13	N	6/27/7	3.5 (3.0–5.0)	
Chen *et al*.^39^	2023	China	RLR	41	53±13	24	39/2	22.5±2.6	1/21/19	5.3±2.2	S7, S8, S4a, S1
			LLR	41	54±12	27	39/2	23.1±2.6	3/21/17	4.8±2.7	
Kato *et al*.^41^	2023	Japan	RLR	91	71 (23–88)	62	81/10	22.9 (15.2–30.7)	0/60/0	2.2 (0.6–1.6)	N
			LLR	91	70 (26–86)	63	79/12	23.0 (16.7–32.4)	0/60/0	2.4 (0.7–1.6)	
Liu *et al*.^43^	2023	MC	RLR	221	61 (52–68)	167	145/76	N	7/209/5	4.5 (3.0, 6.0)	N
			LLR	221	63 (52–70)	172	414/80	N	6/210/5	4.0 (2.7, 7.0)	
Zhang *et al*.^44^	2023	China	RLR	43	48 (26–62)	13	N	22.4 (18.8–32.9)	0/0/43	9 (5.6–2.0)	N
			LLR	86	49 (27–66)	26	N	22.5 (18.3–33)	0/0/86	9 (5–2.5)	
Zhu *et al*.^45^	2023	China	RLR	56	52 (28–72)	45	51/5	23.4 (15.9–30.9)	0/56/0	3.3 (1.0–1.25)	N
			LLR	56	53 (24–72)	47	53/3	23.3 (16.6–31.2)	0/56/0	3.3 (1.1–1.43)	
Kwak *et al*.^42^	2023	MC	RLR	48	62 (53–68)	20	39/9	N	0/0/48	N	N
			LLR	48	63 (47–68)	20	38/10	N	0/0/48	N	
Chong *et al*.^40^	2023	MC	RLR	179	60±14	111	125/54	N	29/102/17	3.0±2.9	Left lateral sectionectomy
			LLR	179	61±17	115	133/46	N	32/101/7	3.0±3.0	
Cheung *et al*.^17^	2023	MC	RLR	73	54 (40–66)	34	51/22	25.0 (22.6–28.5)	8/24/41	11.5 (10.0–13.5)	N
			LLR	219	55 (42–68)	105	159/60	24.0 (21.6–27.3)	28/82/108	11.0 (10.0–13.0)	

ASA, American Society of Anesthesiologists; BMI, body mass index; CRLM, colorectal cancer liver metastases; HCC, hepatocellular carcinoma; MC, multicenter; N, not available; NOS, Newcastle−Ottawa scale; S, sectionectomy.

### Meta-analysis results

A summary of the meta-analysis results is shown in Table 2.

**Table 2:** Results of meta-analysis comparison of LLR and RLR.

Outcomes of interest	Number of studies	Number of patients	*I*^2^ (%)	Model	Overall effect size	95% CI of overall effect size	*P*
Primary outcomes							
Overall complications	21	4908	41	Fixed	OR=0.99	[0.86, 1.14]	0.91
Severe complications	20	4880	0	Fixed	OR=0.76	[0.61, 0.95]	**0.02**
R0	14	3399	0	Fixed	OR=1.28	[1.00, 1.63]	0.05
Secondary outcomes							
Operating time (min)	19	4502	68	Random	SMD=0.07	[−0.05, 0.18]	0.25
Blood loss (ml)	17	3912	84	Random	SMD=−0.31	[−0.48, −0.14]	**0.0005**
Transfusion	17	4350	29	Fixed	OR=0.96	[0.78, 1.19]	0.72
Pringle applied	16	4399	91	Random	OR=0.73	[0.44, 1.22]	0.23
Pringle duration (min)	13	2964	85	Random	SMD=−0.01	[−0.22, 0.19]	0.89
Open conversion	19	4894	45	Fixed	OR=0.46	[0.37, 0.58]	**<0.0001**
Hospital stay (day)	18	4361	62	Random	SMD=−0.02	[−0.13, 0.08]	0.66
Reoperation	14	3914	0	Fixed	OR=0.67	[0.38, 1.18]	0.20
30-day readmission	14	3950	0	Fixed	OR=1.12	[0.83, 1.51]	0.47
30-day mortality	13	3769	0	Fixed	OR=1.11	[0.55, 2.24]	0.77
90-day mortality	15	4545	0	Fixed	OR=0.79	[0.47, 1.34]	0.38

Statistically significant results are shown in bold.

LLR, laparoscopic liver resection; RLR, robotic liver resection; SMD/OR, standard mean difference/odds ratio.

#### Primary outcomes

**Figure 2:**
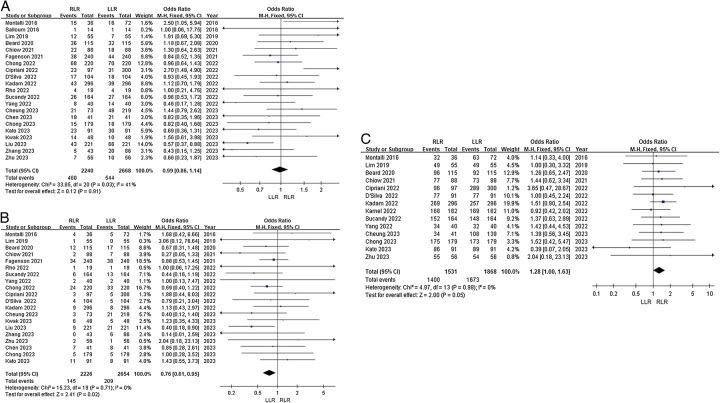
Forest plots of primary outcomes for RLR versus LLR. (A) Overall complications; (B) severe complications; and (C) R0 resection.

*Overall complications*: Twenty-one studies reported overall postoperative complications. Heterogeneity among the studies was not high (*I*^2^=41%). Fixed-effects model analysis showed no significant difference in the odds of overall complications between the groups (OR=0.99, 95% CI 0.86–1.14, *P*=0.91, Fig. 2A).

*Severe complications*: Twenty studies reported severe postoperative complications. Heterogeneity among the studies was low (*I*^2^=0%). Fixed-effects model analysis showed that the odds of severe complications were significantly lower in the RLR group (OR=0.76, 95% CI 0.61−0.95, *P*=0.02, Fig. 2B).

*R0 resection*: Fourteen articles reported R0 resection rates. Heterogeneity among the studies was low (*I*^2^=0%). Fixed-effects model analysis showed no significant difference in odds of R0 resection between the groups (OR=1.28, 95% CI 1.00–1.63, *P*=0.05, Fig. 2C).

#### Secondary outcomes

**Figure 3:**
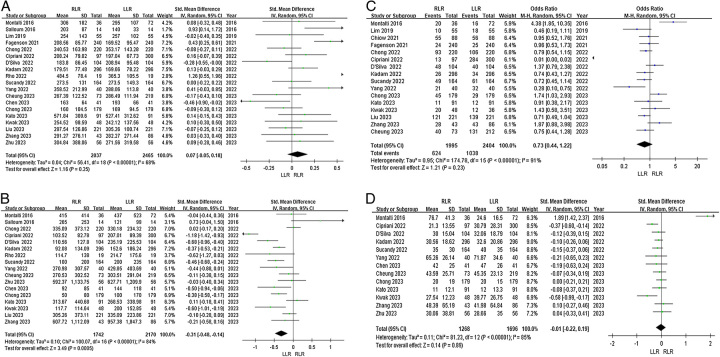
Forest plots of secondary outcomes for RLR versus LLR. (A) Operation time; (B) blood loss; (C) Pringle applied; and (D) duration of Pringle maneuver.

*Operation time*: Nineteen articles reported operation time. Heterogeneity among the studies was high (*I*^2^=68%). Random-effects model analysis showed that operation time did not significantly differ between the RLR and LLR groups (SMD=0.07, 95% CI −0.05 to 0.18, *P*=0.25, Fig. 3A).

*Blood loss*: Seventeen articles reported blood loss. Heterogeneity among the studies was high (*I*^2^=84%). Random-effects model analysis showed that blood loss was significantly lower in the RLR group (SMD=−0.31, 95% CI −0.48 to −0.14, *P*=0.0005, Fig. 3B).

*Pringle maneuver*: Sixteen articles reported the utilization rate of the Pringle maneuver. Heterogeneity among the studies was high (*I*^2^=91%). The random-effects model showed that the odds of Pringle maneuver utilization did not significantly differ between the groups (OR=0.73, 95% CI 0.44–1.22, *P*=0.23, Fig. 3C).

*Duration of Pringle maneuver*: Thirteen articles reported duration of the Pringle maneuver. Heterogeneity among the studies was high (*I*^2^=85%). The random-effects model showed no significant difference in maneuver duration between the groups (SMD=−0.01, 95% CI −0.22 to 0.19, *P*=0.89, Fig. 3D).

**Figure 4:**
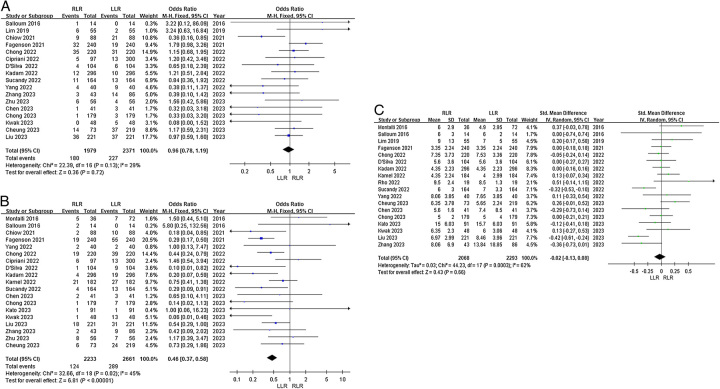
Forest plots of secondary outcomes for RLR versus LLR. (A) Transfusion; (B) open conversion; and (C) postoperative hospital stay.

*Transfusion*: Seventeen articles reported incidence of intraoperative blood transfusion. Heterogeneity among the studies was low (*I*^2^=29%). Fixed-effects model analysis showed that the odds of intraoperative blood transfusion did not significantly differ between the groups (OR=0.96, 95% CI 0.78–1.19, *P*=0.72, Fig. 4A).

*Open conversion*: Nineteen articles reported the rate of open conversion. Heterogeneity among the studies was high (*I*^2^=45%). The fixed-effects model showed that the odds of open conversion were significantly lower in the RLR group (OR, 0.46; 95% CI 0.37–0.58, *P* <0.0001, Fig. 4B).

*Postoperative hospital stay*: Eighteen articles reported length of hospital stay. Heterogeneity among the studies was high (*I*^2^=62%). Random-effects model analysis showed no significant difference in length of hospital stay between the groups (SMD=−0.02, 95% CI −0.13 to 0.08, *P*=0.66, Fig. 4C).

**Figure 5:**
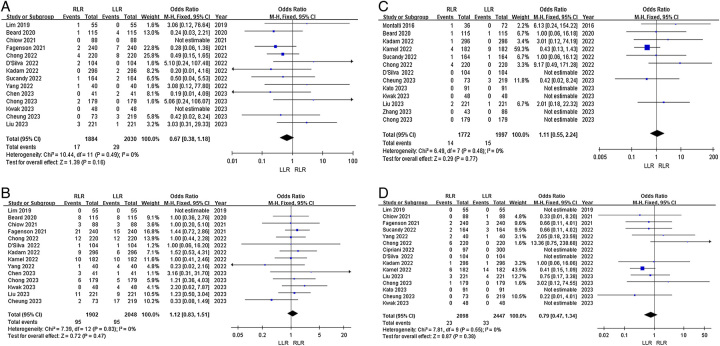
Forest plots of secondary outcomes for RLR versus LLR. (A) Reoperation; (B) 30-day readmission; (C) 30-day mortality; and (D) 90-day mortality.

*Reoperation:* Fourteen articles reported reoperation rates. Heterogeneity among the studies was low (*I*^2^=0%). Fixed-effects model analysis showed no significant difference in odds of reoperation between the groups (OR=0.67, 95% CI 0.38–1.18, *P*=0.20, Fig. 5A).

*30-day readmission*: Fourteen articles reported 30-day readmission rates. Heterogeneity among the studies was low (*I*^2^=0%). Fixed-effects model analysis showed no significant difference in odds of 30-day readmission between the groups (OR=1.12, 95% CI 0.83–1.51, *P*=0.47, Fig. 5B).

*30-day mortality*: Thirteen articles reported 30-day mortality. Heterogeneity among the studies was low (*I*^2^=0%). Fixed-effects model analysis showed no significant difference in odds of 30-day mortality between the groups (OR=1.11, 95% CI 0.55–2.24, *P*=0.77, Fig. 5C).

*90-day mortality*: Fifteen articles reported 90-day mortality. Heterogeneity among the studies was low (*I*^2^=0%). Fixed-effects model analysis showed no significant difference in 90-day mortality between the groups (OR=0.79, 95% CI 0.47–1.34, *P*=0.38, Fig. 5D).

### Subgroup analysis

There was a high degree of heterogeneity in operation time, intraoperative blood loss, use of Pringle maneuver, duration of Pringle maneuver, and length of hospital stay. Studies were divided into subgroups according to date of publication (2023 and before 2023), number of research centers (single and multicenter), and study NOS score (9 and <9). The random-effects model was used for analysis.

In the 2023 publication subgroup, heterogeneity for operation time decreased significantly (*I*^2^=3%) and operation time did not significantly differ between the LLR and RLR groups (SMD= −0.05, 95% CI −0.15 to 0.05, *P*=0.30); moreover, heterogeneity for Pringle maneuver duration decreased significantly (*I*^2^=24%) and maneuver duration did not significantly differ between LLR and RLR (SMD=−0.08, 95% CI −0.22 to 0.05, *P*=0.24). There was no significant change in the heterogeneity of the remaining results (Table 3, Supplemental Figs S1–S2, Supplemental Digital Content 4, http://links.lww.com/JS9/B272; Supplemental Digital Content 5, http://links.lww.com/JS9/B273).

**Table 3:** Results of published year subgroup.

	Published in 2023						Published before 2023					
Subgroup	Number of studies	Number of patients	*I*^2^ (%)	Overall effect size	95% CI of overall effect size	*P*	Number of studies	Number of patients	*I*^2^ (%)	Overall effect size	95% CI of overall effect size	*P*
Operating time (min)	8	1693	3	SMD=−0.05	[−0.15, 0.05]	0.30	11	2809	76	SMD=0.17	[−0.01, 0.34]	0.06
Blood loss (ml)	8	1693	54	SMD=−0.2	[−0.36, −0.05]	**0.01**	9	2219	89	SMD=−0.39	[−0.67, −0.10]	**0.009**
Pringle applied	6	1480	57	OR=1.09	[0.75, 1.61]	0.65	10	2919	94	OR=0.56	[0.25, 1.23]	0.15
Pringle duration (min)	7	1251	24	SMD=−0.08	[−0.22, 0.05]	0.24	6	1713	93	SMD=0.12	[−0.29, 0.52]	0.57
Hospital stay (days)	7	1581	74	SMD=−0.11	[−0.32, 0.10]	0.29	11	2780	41	SMD=0.02	[−0.09, 0.13]	0.69

SMD/OR, standard mean difference/odds ratio; statistically significant results are shown in bold.

In the single-center and multicenter subgroups, heterogeneity for length of hospital stay decreased significantly (*I*^2^=39% and 47%, respectively). Length of hospital stay did not significantly differ between the single-center (SMD=−0.04, 95% CI −0.19 to 0.12, *P*=0.65) and multicenter subgroups (SMD=0.03, 95% CI −0.09 to 0.14, *P*=0.63). There was no significant change in the heterogeneity of the other results (Table 4, Supplemental Figs S3–S4, Supplemental Digital Content 6, http://links.lww.com/JS9/B274; Supplemental Digital Content 7, http://links.lww.com/JS9/B275).

**Table 4:** Results of center subgroup.

	Single center						Multicenter					
Subgroup	Number of studies	Number of patients	*I*^2^ (%)	Overall effect size	95% CI of overall effect size	*P*	Number of studies	Number of patients	*I*^2^ (%)	Overall effect size	95% CI of overall effect size	*P*
Operating time (min)	8	1448	75	SMD=0.23	[−0.01, 0.48]	0.06	11	3054	23	SMD=−0.03	[−0.11, 0.06]	0.56
Blood loss (ml)	7	968	91	SMD=−0.27	[−0.75, 0.21]	0.27	10	2944	71	SMD=−0.3	[−0.45, −0.16]	<**0.01**
Pringle applied	4	1188	98	OR=0.34	[0.03, 3.68]	0.38	12	3211	67	OR=0.93	[0.69, 1.24]	0.61
Pringle duration (min)	5	902	39	SMD=−0.13	[−0.32, 0.06]	0.18	8	2062	90	SMD=0.05	[−0.25, 0.36]	0.74
Hospital stay (days)	7	1307	39	SMD=−0.04	[−0.19, 0.12]	0.65	11	2612	47	SMD=0.03	[−0.09, 0.14]	0.63

SMD/OR, standard mean difference/odds ratio; statistically significant results are shown in bold.

In the NOS score 9 subgroup, heterogeneity for use of Pringle maneuver (*I*^2^=0%) and maneuver duration (*I*^2^=0%) decreased significantly. In addition, the utilization rate was significantly higher in the LLR group than the RLR group (OR= 0.74; 95% CI, 0.60–0.92, *P*=0.006). Duration of Pringle maneuver did not significantly differ between the groups (SMD=−0.11, 95% CI −0.21 to 0.00, *P*=0.90). There was no significant change in the heterogeneity of the other results (Table 5, Supplemental Figs S5–S6, Supplemental Digital Content 8, http://links.lww.com/JS9/B276; Supplemental Digital Content 9, http://links.lww.com/JS9/B277).

**Table 5:** Results of NOS score subgroup.

	NOS=9						NOS<9					
Subgroup	Number of studies	Number of patients	*I*^2^ (%)	Overall effect size	95% CI of overall effect size	*P*	Number of studies	Number of patients	*I*^2^ (%)	Overall effect size	95% CI of overall effect size	*P*
Operating time (min)	7	2324	74	SMD=0.1	[−0.07, 0.28]	0.24	12	2178	68	SMD=0.04	[−0.11, 0.20]	0.62
Blood loss (ml)	7	2241	91	SMD=−0.37	[−0.67, −0.07]	**0.02**	10	1671	72	SMD=−0.26	[−0.46, −0.06]	**0.01**
Pringle applied	6	2205	0	OR=0.74	[0.60, 0.92]	**0.006**	10	2194	95	OR=0.77	[0.31, −1.88]	0.56
Pringle duration (min)	5	1404	0	SMD=−0.11	[−0.21, 0.00]	0.90	8	1560	91	SMD=0.06	[−0.31, 0.43]	0.89
Hospital stay (days)	6	2212	58	SMD=−0.02	[−0.16, 0.12]	0.78	12	2149	66	SMD=−0.02	[−0.18, 0.14]	0.80

NOS, Newcastle−Ottawa scale; SMD/OR, standard mean difference/odds ratio; statistically significant results are shown in bold.

### Sensitivity analysis and publication bias

**Figure 6:**
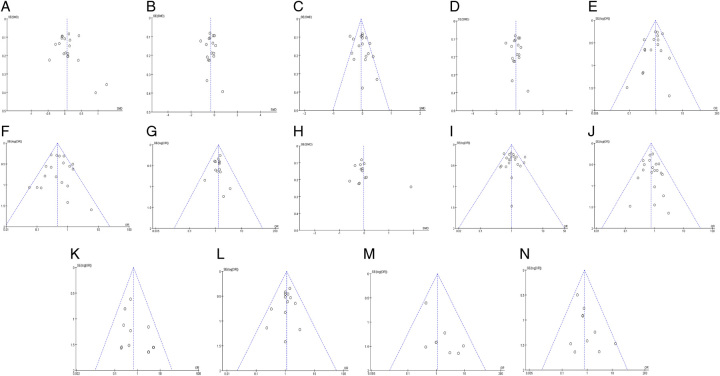
Funnel plot of RLR versus LLR. (A) Operation time; (B) blood loss; (C) Pringle applied; (D) duration of Pringle maneuver; (E) transfusion; (F) open conversion; (G) R0; (H) postoperative hospital stay; (I) overall complications; (J) severe complications; (K) reoperation; (L) 30-day readmission; (M) 30-day mortality; and (N) 90-day mortality.

The results regarding operation time, intraoperative blood loss, use and duration of Pringle maneuver, and length of postoperative hospital stay were highly heterogeneous. Excluding the included literature one by one, the intraoperative blood loss heterogeneity had no significant change, suggesting that the analysis results were stable. After excluding the studies by Fagenson *et al*.^32^ and Rho *et al*.^36^ from the operation time data, the heterogeneity was significantly lower (*I*^2^=38%); repeat analysis using the fixed-effects model showed no significant difference in operation time between the two groups (SMD=0.00, 95% CI −0.06 to 0.06, *P*=0.98). After excluding Montalti *et al*.^28^ and Cipriani *et al*.^33^ from the Pringle maneuver use data, the heterogeneity was significantly lower (*I*^2^=47%); repeat analysis using the fixed-effects model still showed no significant difference in odds of Pringle maneuver use between the groups (OR=0.88, 95% CI 0.76–1.02, *P*=0.09). After excluding Montalti *et al*.^28^ from the Pringle maneuver duration data, the heterogeneity was significantly lower (*I*^2^=18%); repeat analysis using the fixed-effects model showed that the duration was significantly longer in the LLR group (SMD=−0.13, 95% CI −0.21 to −0.05, *P*=0.0008). After excluding Liu *et al*.^43^ from length of postoperative hospital stay, the heterogeneity was significantly lower (*I*^2^=41%); repeat analysis using the fixed-effects model showed no significant difference in length of postoperative hospital stay between the groups (SMD=0.00, 95% CI −0.07 to 0.06, *P*=0.94). A funnel chart was constructed based on the analysis results, and the publication bias test showed that the distribution of scatter spots on both sides of the funnel was basically symmetrical, suggesting that there was no obvious publication bias (Fig. 6).

### Meta-regression analysis and quality of evidence

Meta-regression analysis showed that date of publication (*P*=0.059), sample size (*P*=0.051), male-to-female ratio (*P*=0.467), age (*P*=0.477), ASA grade (*P*=0.928), and study NOS score (*P*=0.113) had no significant effect on incidence of all complications, incidence of severe complications, nor R0 resection rate. The regression analysis results are summarized in Supplemental Tables S5–S7 (Supplemental Digital Content 3, http://links.lww.com/JS9/B271). The quality of evidence for overall and severe complications was moderate, while that for R0 resection rate was low. The quality of evidence grade of secondary outcome index was moderate in intraoperative blood transfusion, conversion to laparotomy, and reoperation, operation time, intraoperative bleeding, Pringle blocking rate, 30-day readmission, 30-day mortality, and 90-day mortality were low. The quality of evidence for duration of Pringle maneuver was very low. The quality of evidence data is summarized in Supplemental Figure S7 (Supplemental Digital Content 10, http://links.lww.com/JS9/B278).

## Discussion

This meta-analysis of 22 propensity score-matched studies showed that RLR and LLR have similar operation times, durations of Pringle maneuver, intraoperative blood transfusion rates, lengths of hospital stay, and incidence of overall complications. However, RLR appears to be superior in terms of less blood loss, a lower open conversion rate, and a lower incidence of severe complications. RLR appears to be a safe and effective minimally invasive alternative to LLR.

The concept of minimally invasive surgery (MIS) was first proposed in 1983^46^. At its core is less trauma, less intraoperative bleeding, shorter hospital stay, and faster postoperative recovery^47^. Laparoscopic surgery is minimally invasive and has been demonstrated in RCTs to be safe and feasible for abdominal operations^48–50^. However, it is associated with several problems, including lack of three-dimensional visualization, tremor effect, limitations related to instrumentation angles, and others. Robotic surgical platforms seem able to overcome some of these shortcomings. However, whether robotic surgery is superior to laparoscopic surgery remains debatable. Several RCTs have attempted to answer the question. Feng *et al*.^51^ suggested that robotic surgery is superior to laparoscopic for middle and low rectal cancers. In a distal gastrectomy study, Lu *et al*.^52^ suggested that robotic surgery is superior. For abdominal hernia repair, robotic and laparoscopic surgery appear to be equivalent^53–55^. These studies show that robotic and laparoscopic surgery have differences that vary according to the type of operation. A RCT comparing robotic and laparoscopic liver surgery has not yet been conducted, so there is currently a lack of high-level evidence to support the merits of RLR.

An early small study reported fewer complications and shorter hospital stay after robotic hepatectomy. Another suggested that outcomes were similar but the cost of robotic surgery was higher^56,57^. A meta-analysis of nine studies by Qiu *et al*.^58^ reported that RLR is more expensive and associated with longer operation time. Another 14 studies by Rahimli *et al*.^22^ reported that RLR and LLR outcomes were similar. It is well known that male:female ratio, age, type of disease, tumor size, extent of hepatectomy, and other factors have an effect on liver surgery outcome. At present, the published meta-analysis of RLR and LLR does not seem to match the preoperative scores of the above factors, resulting in higher heterogeneity and lower level of evidence.

To the best of our knowledge, this is the first meta-analysis of propensity score-matched studies to compare short-term outcomes between RLR and LLR. Operation time did not differ. Surgical proficiency is a crucial determinant of operation time, and operation times are generally longer for surgeons who are less experienced with a procedure. Kim *et al*.^59^ argued that the learning curve for RLR requires 16 cases, which is consistent with the 15 cases suggested by Chen *et al*.^60^. In our meta-analysis, only Salloum *et al*.^29^ had a sample size of less than 15 cases. Our results suggest that operation times are equivalent after surgeons have gained sufficient experience in the procedure. Intraoperative blood loss is another important outcome that affects hepatectomy prognosis. Use of the Pringle maneuver and its duration of application affect the amount of bleeding during hepatectomy, which is reflected by intraoperative blood transfusion volume^61–63^. We found no difference in use of the Pringle maneuver between RLR and LLR. However, in the LLR, maneuver duration was longer and intraoperative blood loss was lower. Intraoperative blood transfusion volume did not differ between the groups. RLR enables three-dimensional visualization, tremor filtering, and higher degrees of freedom for the instruments, which makes the operation more precise and stable. As a result, intraoperative blood loss was lower.

The most significant finding of our meta-analysis was lower odds of open conversion for RLR (OR=0.46, *P* <0.0001). Conversion to laparotomy increases the risk of postoperative complications^64^ and affects long-term outcome in patients undergoing hepatectomy^65^. Abdominal adhesions, tumor size and location, intraoperative bleeding, and technical operation were the reasons for the transition between RLR and LLR^66,67^. In our meta-analysis, confounding was reduced using propensity score matching. Previous studies also found that intraoperative bleeding was less with RLR. This indicates that the technical advantages of RLR reduces the risk of conversion to laparotomy during operation. These advantages also seem to reduce the odds of severe postoperative complications (OR=0.76, *P*=0.02). Although we found no difference in the odds of overall complications between RLR and LLR, the odds of severe complications were lower for RLR, which may also be the potential benefit point for the lower conversion rate of RLR to laparotomy. However, whether the lower odds of open conversion associated with RLR affect long-term outcome has not been discussed in depth. In addition, although the odds of severe complications for LLR were higher, this did not affect length of hospital stay or odds of reoperation, 30-day readmission, 30-day mortality, or 90-day mortality. We found no difference between LLR and RLR in any of these indicators, which shows that LLR is certainly a safe operation. Furthermore, short-term outcomes were only partially better with RLR, and associated costs are higher. The high cost of RLR infrastructure will probably limit the application of RLR^68,69^.

Our meta-analysis has several limitations. First, it does not include any RCTs. Second, there are differences between studies in the extent and location of resection (segmental vs. hepatectomy), texture of the liver, type of liver tumor, robotic and laparoscopy systems used, and patient characteristics such as sex and age that increased the heterogeneity of the results. Finally, data overlap between multicenter studies may have introduced bias. Future multicenter RCTs are needed to validate the differences between RLR and LLR.

In summary, the results of this meta-analysis of propensity score-matched studies show that although some short-term outcomes are similar between RLR and LLR, RLR is superior in terms of less blood loss and lower odds of open conversion and severe complications. In the future, RLR may become a safe and effective replacement for LLR; however, further study is needed.

## Ethical approval

The study was approved by the International prospective register of systematic reviews (PROSPERO).

## Sources of funding

This work was supported by grants from the National Natural Science Foundation of China (82173124, 82103533, 81972747, and 82203823), the Natural Science Foundation of Sichuan Province (2023NSFSC1877), and the Science and Technology Program of Sichuan Province (2023JDR0077).

## Author contribution

F.G.: conceptualization, methodology, resources, writing – original draft, review, and editing; X.Z.: conceptualization, methodology, resources, writing – original draft, review, and editing; K.J.: investigation, formal analysis, writing – original draft and editing, and supervision; Q.X.: investigation, formal analysis, and writing – original draft and editing; M.Y.: investigation, supervision, and writing; T.M.: investigation, and supervision; H.W.: project administration, investigation, supervision, and writing.

## Conflicts of interest disclosure

No conflicting relationship exists for any of the authors.

## Research registration unique identifying number (UIN)

1. Name of the registry used: PROSPERO.
2. Unique identifying number or registration ID: CRD42023417941.
3. Hyperlink to your specific registration (must be publicly accessible and will be checked): https://www.crd.york.ac.uk/prospero/display_record.php?ID=CRD42023417941.

## Guarantor

Pro. Hong Wu, E-mail: wuhong@scu.edu.cn; Liver Transplantation Center, No. 37, Guoxue Lane, Wuhou District, Chengdu, Sichuan Province, China.

## Date availability statement

All data generated or analyzed during this study are included in this article. The data are available from the corresponding author upon reasonable request.

## Supplementary Material
