# Supplementary material for: Comparison of short-term outcomes between robotic and laparoscopic liver resection: a meta-analysis of propensity score-matched studies
Source: Int J Surg. 2023 Nov 3;110(2):1126–38. doi: 10.1097/JS9.0000000000000857 (PMC10871648; doi:10.1097/JS9.0000000000000857)
Supplement: Supplementary file 1 [file js9-110-1126-s001.docx]

**Supplementary table S1 - Summary of search strategies for Pubmed**

|  | *Search strategies (Pubmed)* |  |
| --- | --- | --- |
| #1 | Laparoscopy [Title/Abstract]/ | 46160 |
| #2 | Laparoscopic [Title/Abstract]/ | 129575 |
| #3 | Laparoscope [Title/Abstract]/ | 2309 |
| #4 | #1 or #2 or #3 | 149,355 |
| #5 | Robot [Title/Abstract]/ | 29,601 |
| #6 | robot-assisted [Title/Abstract]/ | 12,795 |
| #7 | Robotic [Title/Abstract]/ | 42,598 |
| #8 | robotic-assisted [Title/Abstract]/ | 7,476 |
| #9 | computer-assisted surgery [Title/Abstract]/ | 1,687 |
| #10 | da Vinci [Title/Abstract]/ | 4,147 |
| #11 | #5 or #6 or #7 or #8 or #9 or #10 | 62,028 |
| #12 | #4 and #11 | 13,051 |
| #13 | liver resection [Title/Abstract]/ | 12,474 |
| #14 | Hepatectomy [Title/Abstract]/ | 25,503 |
| #15 | Sectionectomy [Title/Abstract]/ | 527 |
| #16 | Hemihepatectomy [Title/Abstract]/ | 1,250 |
| #17 | Lobectomy [Title/Abstract]/ | 21,872 |
| #18 | #13 or #14 or #15 or #16 or #17 | 55,849 |
| #19 | #12 and #18 | 436 |

**Supplementary table S2 - Summary of search strategies for EMBASE**

|  | *Search strategies (EMBASE)* |  |
| --- | --- | --- |
| #1 | laparoscopy:ti,ab,kw/ | 68,515 |
| #2 | laparoscopic:ti,ab,kw/ | 201074 |
| #3 | laparoscope:ti,ab,kw/ | 3433 |
| #4 | #1 or #2 or #3 | 224,436 |
| #5 | robot:ti,ab,kw/ | 39,360 |
| #6 | robot-assisted:ti,ab,kw/ | 20,117 |
| #7 | robotic:ti,ab,kw/ | 67,080 |
| #8 | robotic-assisted:ti,ab,kw/ | 14,926 |
| #9 | computer-assisted surgery:ti,ab,kw/ | 2,239 |
| #10 | da Vinci:ti,ab,kw/ | 7,317 |
| #11 | #5 or #6 or #7 or #8 or #9 or #10 | 90,592 |
| #12 | #4 and #11 | 25,594 |
| #13 | liver resection:ti,ab,kw/ | 19,338 |
| #14 | hepatectomy:ti,ab,kw/ | 30,675 |
| #15 | sectionectomy:ti,ab,kw/ | 1,096 |
| #16 | hemihepatectomy:ti,ab,kw/ | 1,895 |
| #17 | lobectomy:ti,ab,kw/ | 26,708 |
| #18 | #13 or #14 or #15 or #16 or #17 | 70,165 |
| #19 | #12 and #18 | 806 |

**Supplementary table S3 - Summary of search strategies for Cochrane Library**

|  | *Search strategies (Cochrane Library)* |  |
| --- | --- | --- |
| #1 | liver resection [ti,ab,kw] (word variations has been searched)/ |  |
| #2 | hepatectomy [ti,ab,kw] (word variations has been searched)/ |  |
| #3 | sectionectomy [ti,ab,kw] (word variations has been searched)/ |  |
| #4 | hemihepatectomy [ti,ab,kw] (word variations has been searched)/ |  |
| #5 | lobectomy [ti,ab,kw] (word variations has been searched)/ |  |
| #6 | #1 or #2 or #3 or #4 or #5 | 66 |
