# Supplementary material for: Comparison of short-term outcomes between robotic and laparoscopic liver resection: a meta-analysis of propensity score-matched studies
Source: Int J Surg. 2023 Nov 3;110(2):1126–38. doi: 10.1097/JS9.0000000000000857 (PMC10871648; doi:10.1097/JS9.0000000000000857)
Supplement: Supplementary file 2 [file js9-110-1126-s002.docx]

| Table S4 Assessment of the quality of the PSM studies based on the Newcastle–Ottawa Scale | | | | | | | | | |
| --- | --- | --- | --- | --- | --- | --- | --- | --- | --- |
| Study | Selection (Out of 4) | | | | Comparability  (Out of 2) | Outcomes (Out of 3) | | | Total  (Out of 9) |
|  | (1) | (2) | (3) | (4) |  | (5) | (6) | (7) |  |
| Montalti ^[28]^ | * | * | * | * | * | * |  |  | 6 |
| Salloum ^[29]^ | * | * | * | * | * |  |  | * | 6 |
| Lim ^[30]^ | * | * | * | * | * | * | * |  | 7 |
| Beard ^[31]^ | * | * | * | * | ** | * |  | * | 8 |
| Chiow ^[15]^ | * | * | * | * | ** | * | * |  | 8 |
| Fagenson ^[32]^ | * | * | * | * | ** | * | * | * | 9 |
| Chong ^[14]^ | * | * | * | * | ** | * | * | * | 9 |
| Cipriani ^[33]^ | * | * | * | * | * | * |  | * | 7 |
| D'Silva ^[16]^ | * | * | * | * | ** | * | * |  | 8 |
| Kadam ^[34]^ | * | * | * | * | ** | * | * | * | 9 |
| Kamel ^[35]^ | * | * | * | * | * | * |  |  | 6 |
| Rho ^[36]^ | * | * | * | * | * | * |  |  | 6 |
| Sucandy ^[37]^ | * | * | * | * | ** | * | * | * | 9 |
| Yang ^[38]^ | * | * | * | * | ** | * | * | * | 9 |
| Chen ^[39]^ | * | * | * | * | ** | * |  |  | 7 |
| Kato ^[41]^ | * | * | * | * | ** | * | * |  | 8 |
| Liu ^[43]^ | * | * | * | * | ** | * |  | * | 8 |
| Zhang ^[44]^ | * | * | * | * | * | * |  | * | 7 |
| Zhu ^[45]^ | * | * | * | * | ** | * | * | * | 9 |
| Kwak ^[42]^ | * | * | * | * | ** | * |  | * | 8 |
| Chong ^[40]^ | * | * | * | * | ** | * | * |  | 8 |
| Cheung ^[17]^ | * | * | * | * | ** | * | * | * | 9 |
| 1. representativeness of the exposed cohort; 2. selection of the non-exposed cohort; 3. ascertainment of exposure; 4. demonstration that outcome of interest was not present at the start of the study; 5. assessment of outcome; 6. was follow-up long enough for outcomes to occur;   (7) adequacy of follow-up of cohorts. | | | | | | | | | |
