# Supplementary material for: Comparison of short-term outcomes between robotic and laparoscopic liver resection: a meta-analysis of propensity score-matched studies
Source: Int J Surg. 2023 Nov 3;110(2):1126–38. doi: 10.1097/JS9.0000000000000857 (PMC10871648; doi:10.1097/JS9.0000000000000857)
Supplement: Supplementary file 3 [file js9-110-1126-s003.docx]

| Table S5 Results of meta regression analysis for overall complications | | | | |
| --- | --- | --- | --- | --- |
| Variances | Coefficient standard | Standard error | P value | 95% confidence interval |
| Publication year | -0.0854 | 0.0413 | 0.059 | [-0.1747, 0.0039] |
| Sample size | -1.1701 | 0.5437 | 0.051 | [-2.3446, 0.0045] |
| Male/Female | 0.4591 | 0.6097 | 0.467 | [-0.8828, 1.8010] |
| Age | 2.6011 | 3.5342 | 0.477 | [-5.1778, 10.3800] |
| ASA(I-II/III-IV) | -0.0475 | 0.5176 | 0.928 | [-1.1658, 1.0707] |
| NOS | 0.1966 | 0.1142 | 0.113 | [-0.0547, 0.4479] |

ASA, American society of anesthesiologists; NOS, Newcastle-Ottawa.

Table S6 Results of meta regression analysis for severe complications

| Variances | Coefficient standard | Standard error | P value | 95% confidence interval |
| --- | --- | --- | --- | --- |
| Publication year | -0.0228 | 0.0869 | 0.798 | [-0.2121, 0.1665] |
| Sample size | -0.8614 | 1.2639 | 0.508 | [-3.6152, 1.8923] |
| Male/Female | 0.022 | 0.2406 | 0.929 | [-0.5075, 0.5516] |
| Age | 0.3496 | 1.4598 | 0.815 | [-2.8633, 3.5625] |
| ASA(I-II/III-IV) | 0.3772 | 1.2197 | 0.762 | [-2.2803, 3.0347] |
| NOS | 0.002 | 0.0436 | 0.964 | [-0.0940, 0.0981] |

ASA, American society of anesthesiologists; NOS, Newcastle-Ottawa.

Table S7 Results of meta regression analysis for R0

| Variances | Coefficient standard | Standard error | P value | 95% confidence interval |
| --- | --- | --- | --- | --- |
| Publication year | -0.0074 | 0.0096 | 0.463 | [-0.0297, 0.0148] |
| Sample size | -0.0413 | 0.0607 | 0.516 | [-0.1812, 0.0987] |
| Male/Female | 0.0299 | 0.1234 | 0.813 | [-0.2416, 0.3015] |
| Age | -0.3787 | 0.6717 | 0.584 | [-1.8572, 1.0997] |
| ASA(I-II/III-IV) | 0.0252 | 0.0468 | 0.604 | [-0.0827, 0.1331] |
| NOS | -0.0034 | 0.0315 | 0.917 | [-0.0727, 0.6601] |

ASA, American society of anesthesiologists; NOS, Newcastle-Ottawa.
