# Supplementary figures and images for: Comparison of short-term outcomes between robotic and laparoscopic liver resection: a meta-analysis of propensity score-matched studies
Source: Int J Surg. 2023 Nov 3;110(2):1126–38. doi: 10.1097/JS9.0000000000000857 (PMC10871648; doi:10.1097/JS9.0000000000000857)

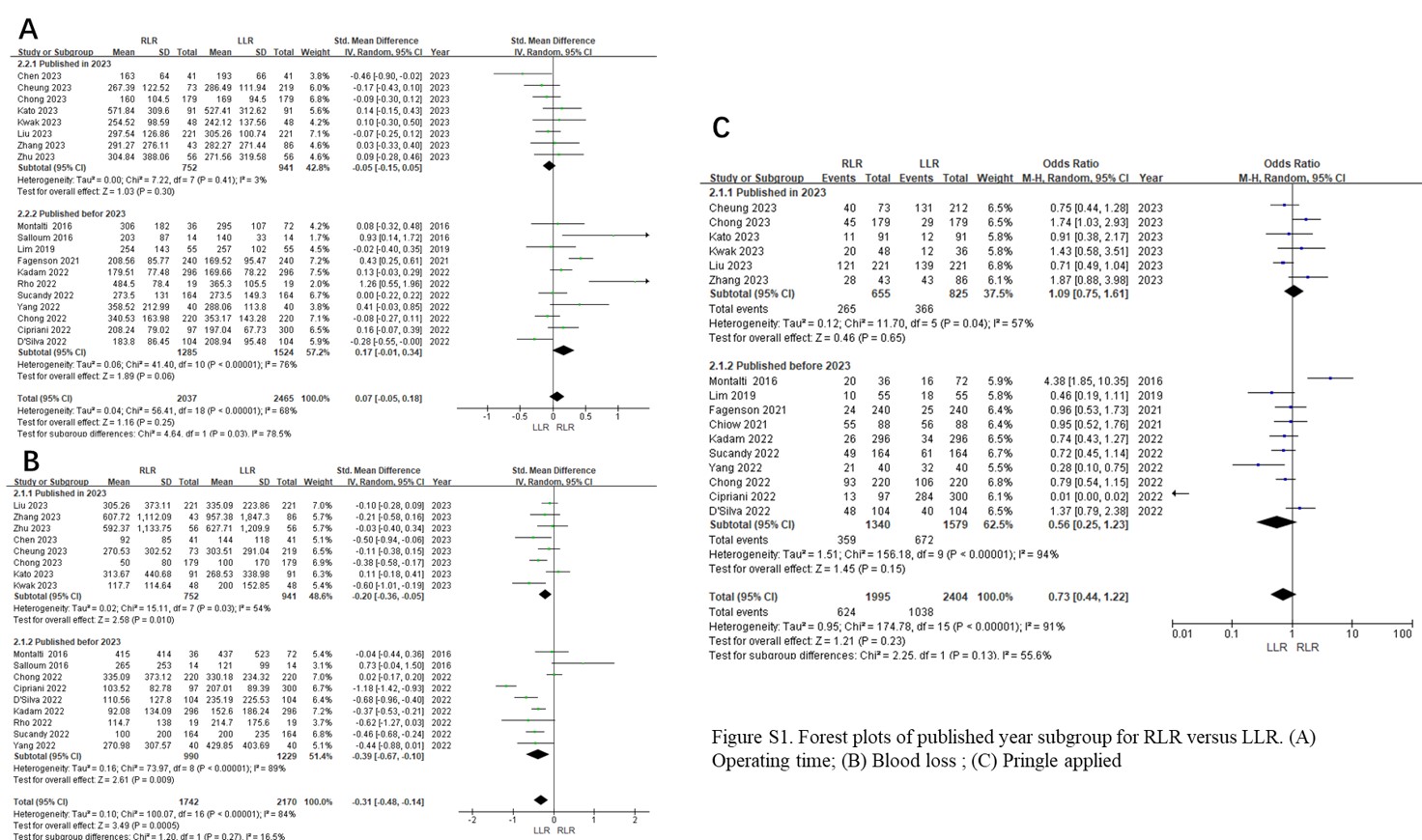

Supplement: Supplementary file 4 [file js9-110-1126-s004.jpg]

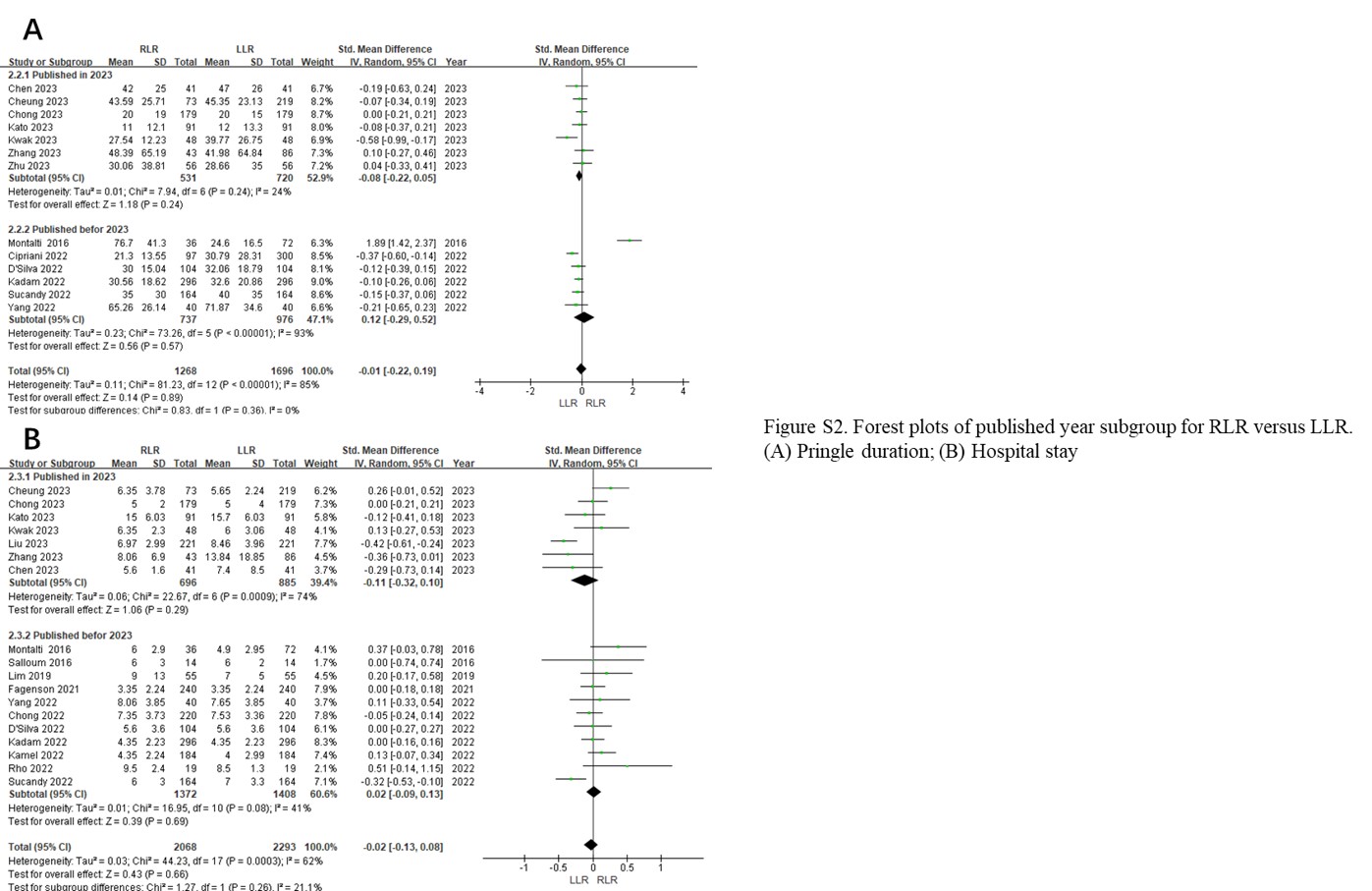

Supplement: Supplementary file 5 [file js9-110-1126-s005.jpg]

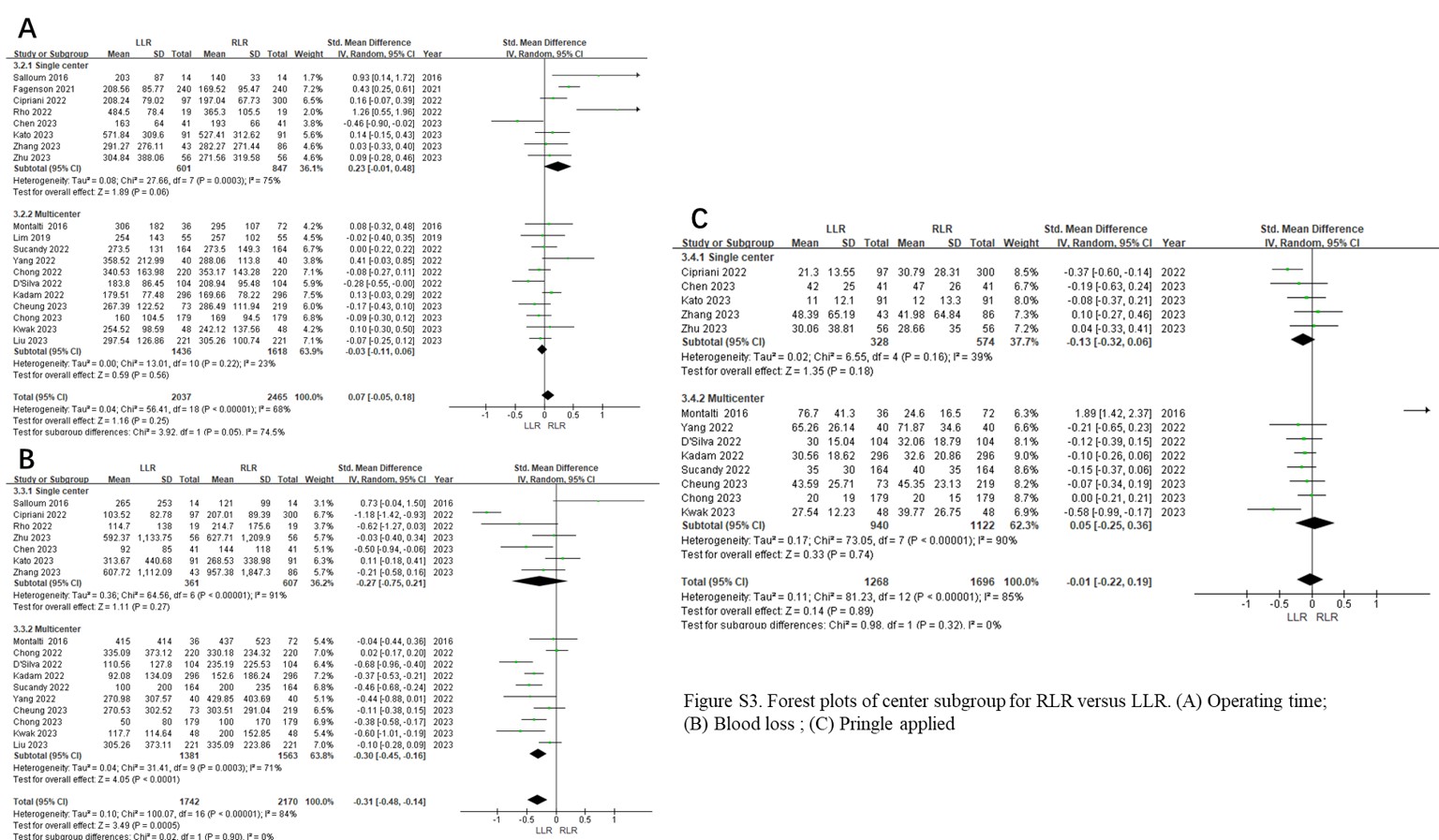

Supplement: Supplementary file 6 [file js9-110-1126-s006.jpg]

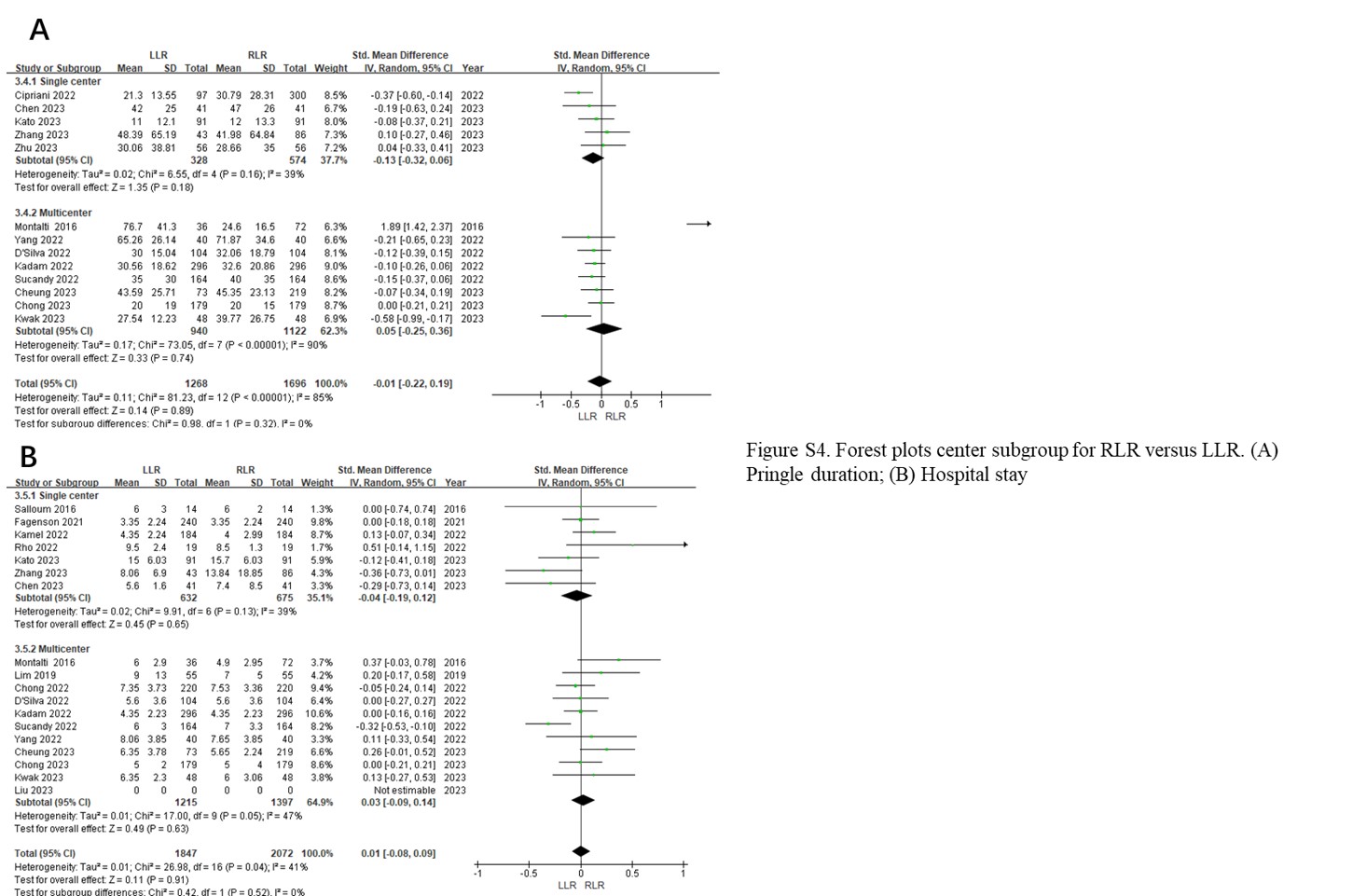

Supplement: Supplementary file 7 [file js9-110-1126-s007.jpg]

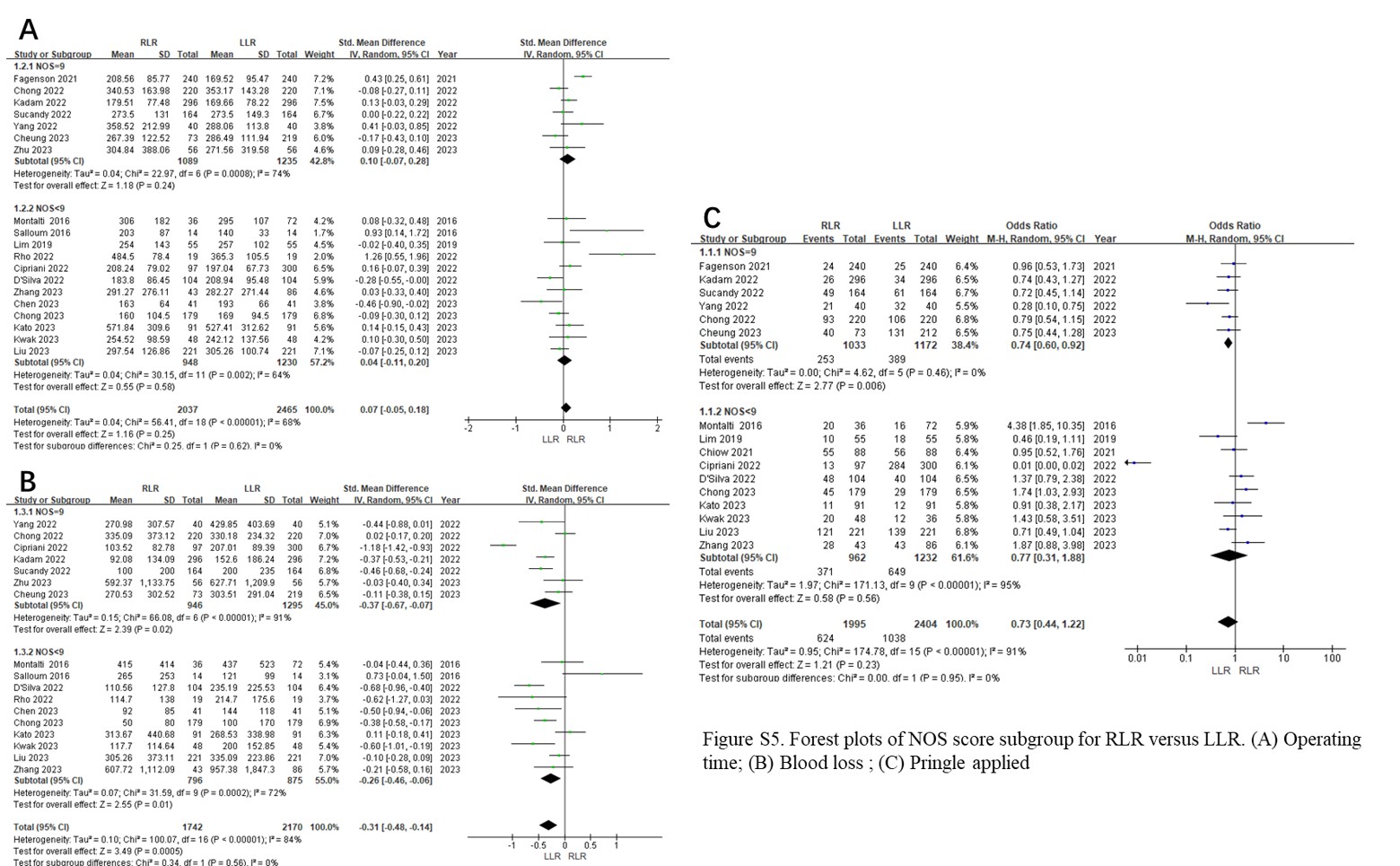

Supplement: Supplementary file 8 [file js9-110-1126-s008.jpg]

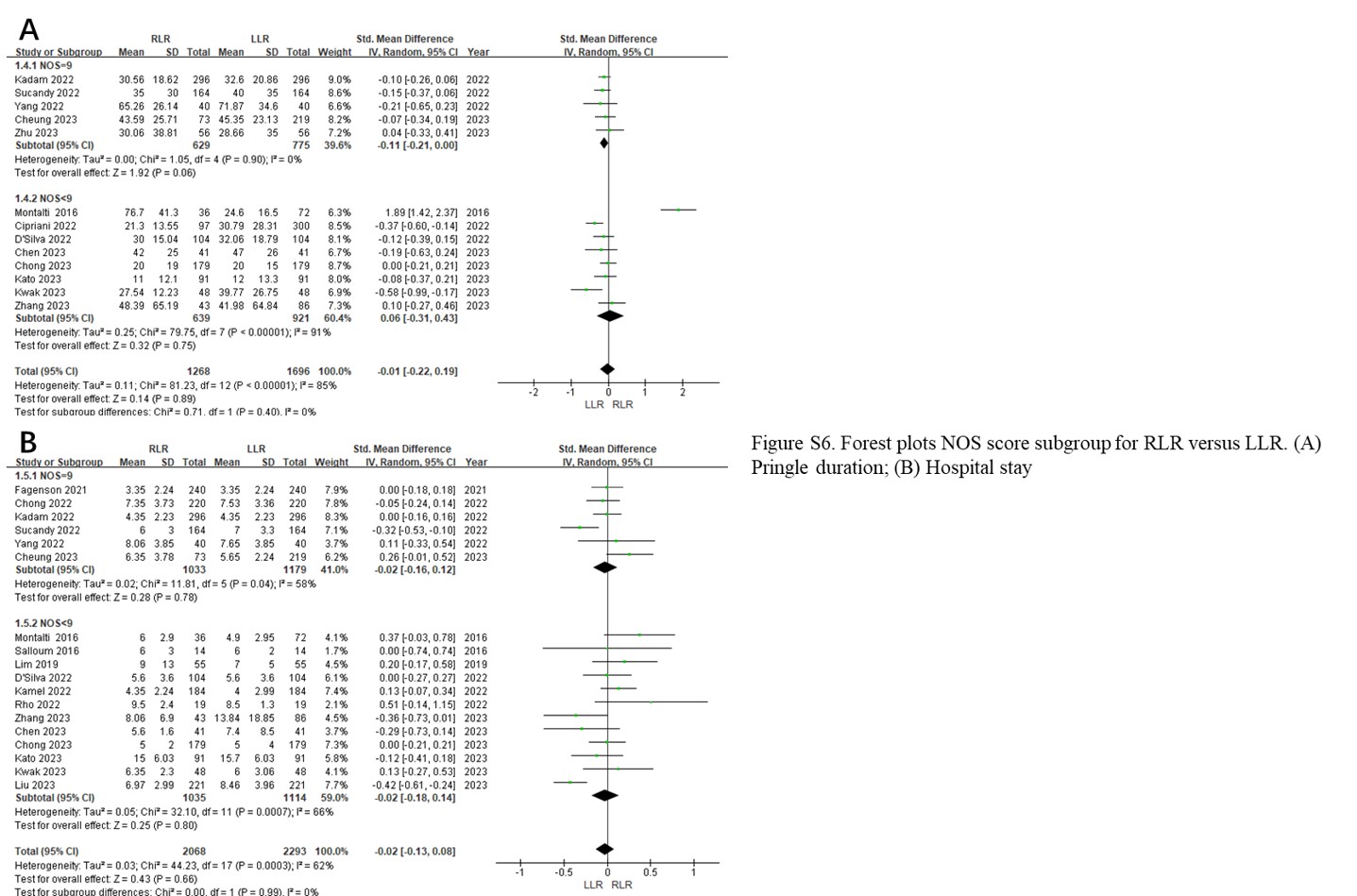

Supplement: Supplementary file 9 [file js9-110-1126-s009.jpg]

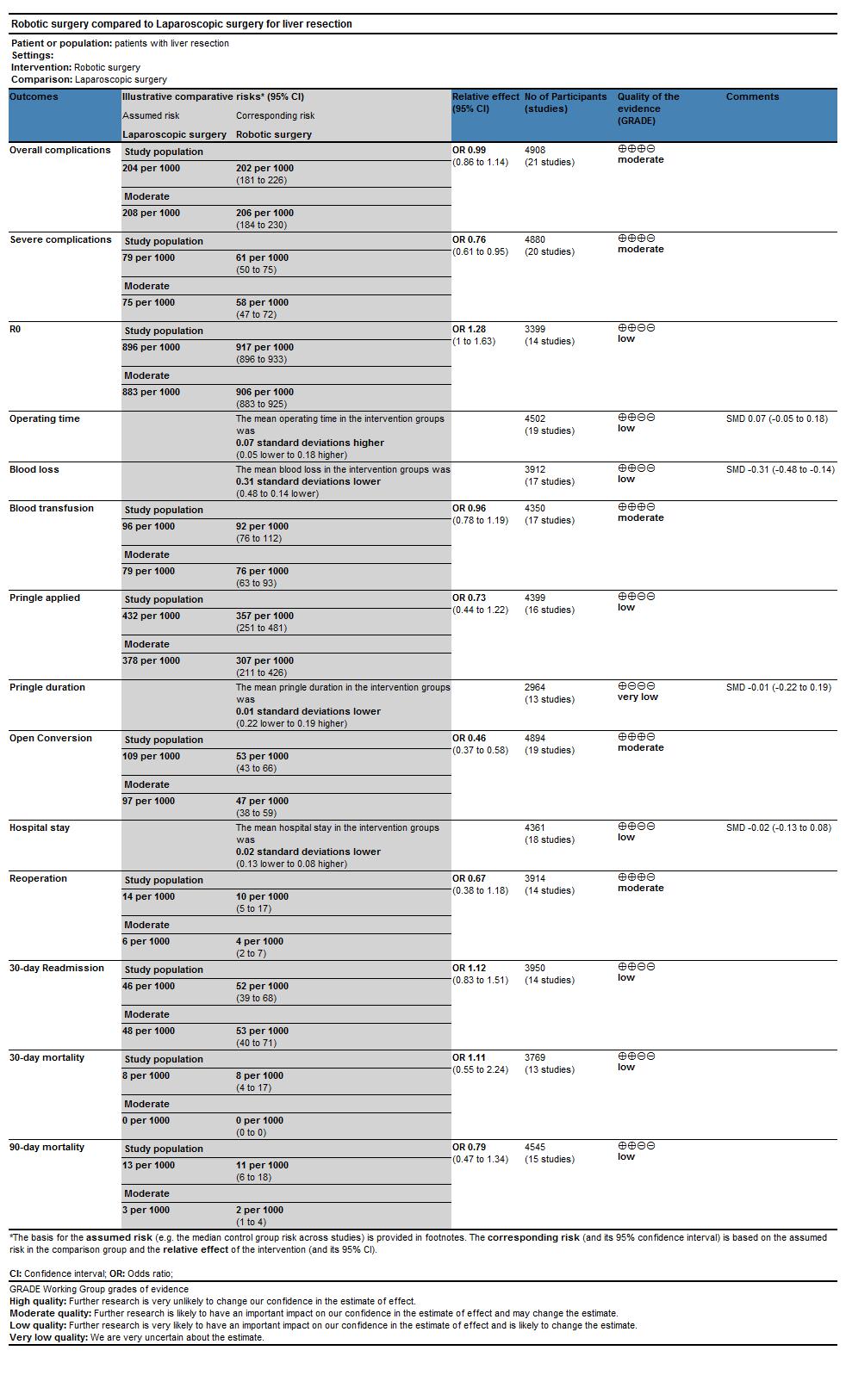

Supplement: Supplementary file 10 [file js9-110-1126-s010.jpeg]
